# Hantavirus Infection in Istanbul, Turkey

**DOI:** 10.3201/eid1702.100663

**Published:** 2011-02

**Authors:** Oral Oncul, Yunus Atalay, Yalcin Onem, Vedat Turhan, Ali Acar, Yavuz Uyar, Dilek Y. Caglayik, Sezai Ozkan, Levent Gorenek

**Affiliations:** Author affiliations: Gulhane Military Medical Academy Haydarpasa Training Hospital, Istanbul, Turkey (O. Oncul, Y. Atalay, Y. Onem, V. Turhan, A. Acar, S. Ozkan, L. Gorenek);; Refik Saydam National Public Health Agency, Ankara, Turkey (Y. Uyar, D.Y. Caglayik)

**Keywords:** Hantavirus, hemorrhagic renal syndrome, Dobrava virus, viruses, global climatic change, viral hemorrhagic fever, Turkey, letter

**To the Editor:** More than 20 serotypes of hantavirus have been identified, and 11 infect humans. Puumala virus (PUUV), Dobrava virus (DOBV), and Seoul virus cause different forms of hemorrhagic renal syndrome (*1**,**2*). DOBV is endemic to Turkey and countries in the Balkan region. Approximately 10,000–12,000 cases of infection with PUUV and DOBV occur in European Russia each year (*3*). Initial case reports identified a hantavirus epidemic (laboratory confirmed) in February 2009 that involved 12 persons in Bartin and Zonguldak in western Turkey near the Black Sea. The hantavirus responsible for this epidemic was a PUUV subtype (*4*). We report a man infected with DOBV in Turkey who died 2 days after admission to an intensive care unit (ICU).

The patient was a 22-year-old man who lived near Istanbul, Turkey. He was admitted to the Silivri State Hospital in March 2010 because of fatigue, diffuse pain, nausea, and vomiting. Approximately 2 hours after admission, ecchymotic rashes developed on his upper extremities and spread to other areas. His general condition worsened, and 15 hours later, he was transferred to the ICU of the Emergency Service of Gulhane Military Medical Academy Haydarpasa Training Hospital. His medical history did not include exposure to rodents or any travel.

At admission to the ICU, his general condition was poor, and his speech was garbled and incoherent. He had a body temperature of 37.2°C, a pulse of 140 beats/min, an arterial blood pressure of 90/60 mm Hg, diffuse hemorrhagic foci, and a disseminated ecchymotic rash. Laboratory test results showed the following: 13,200 leukocytes/mm^3^, 92% polymorphonuclear leukocytes, hemoglobin 11.6 mg/dL, 385,000 platelets/mm^3^, alanine aminotransferase 62 IU/mL, aspartate aminotransferase 170 IU/mL, creatine phosphokinase 2,115 IU/L, lactate dehydrogenase 1,109 IU/L, urea 65 mg/dL, creatinine 3.78 mg/dL, prothrobin time 24.8 s, activated partial thromboplastin time 116.3 s, potassium 2.9 mEq/L, C-reactive protein 326 mg/dL, and erythrocyte sedimentation rate 132 mm/h.

Subsequently, urinary output decreased and respiratory functions worsened. He then lost consciousness and was subjected to mechanic ventilation. Lumbar puncture was not performed because of risk for bleeding (high international normalized ratio values for blood coagulation and thrombocytopenia). Cranial computed tomographic scan did not show any pathologic changes. Treatment with ceftriaxone, 4 g/day intravenously, was initiated, and the dose was adjusted according to creatinine clearance because of suspected meningococcemia. A single dose of prednisolone, 80 mg intravenously, was given concomitantly. Bacterial growth was not observed in cultures of urine and blood samples.

The Hanta Profile 1 EUROLINE Test (Euroimmun, Luebeck, Germany) was used to detect immunoglobulin (Ig) G and IgM against 3 hantavirus serotypes (PUUV, DOBV, and Hantaan virus). Results of a hantavirus IgM immunoblot test were positive for DOBV. The QIAamp viral RNA Mini Extraction Kit (QIAGEN, Hilden, Germany) was used for extraction of viral RNA. PUUV and DOBV RNA in serum and urine samples were investigated by using an in-house real-time PCR (Rotorgene; QIAGEN). DOBV RNA was detected in urine samples by PCR (Table).

**Table:** Detection of Dobrava virus in 22-year-old patient, Turkey*

Test	Hantavirus immunoblot			Real-time PCR				
				Serum			Urine	
	IgM	IgG		DOBV	PUUV		DOBV	PUUV
Result	Pos	Neg		Neg	Neg		Pos	Neg

*Ig, immunoglobulin; DOBV, Dobrava virus; PUUV, Puumala virus; pos, positive; neg; negative.

Meningococcemia, acute hemorrhagic fever, and Crimean-Congo hemorrhagic fever were considered in the differential diagnosis for the patient. Other diseases were excluded by biochemical, serologic, and microbiologic test results. Hantavirus infection was diagnosed in this patient on the basis of criteria recommended by the European Network for Diagnostics of Imported Viral Diseases (*5*). On the second day of treatment, the patient died of cardiopulmonary arrest.

The patient had worked as a security guard in a new prison located in an area that had contained oak and hornbeam forests. DOBV is carried by rodents (*Apodemus flavicollis*), and the habitat of this rodent in Europe is open oak or beech forest. In a field study performed in rural areas of Turkey near the Black Sea and Aegean Sea, hantavirus was detected in *Microtus* spp. voles (*6*). In another study performed in regions near the Aegean Sea, DOBV was detected in 7 (3.5%) of 200 patients with acute or chronic renal failure (*7*). However, information about specific regions in Turkey in which hantavirus is endemic is limited.

Hantavirus infections, which were first identified in northwestern Turkey in 2009 and subsequently in Istanbul, should be considered in the diagnosis of patients who have fever and bleeding. Because of recent emergence of hantavirus in Turkey, areas to which this virus is endemic and where risk for infection is highest have not been identified. Therefore, all inhabitants at high risk for infection (forest workers, military personnel, farmers, persons living in or near a forest, persons handling wood) should be informed about this risk.
